# Nonsurgical Korean Integrative Treatments for Symptomatic Lumbar Spinal Stenosis: A Three-Armed Randomized Controlled Pilot Trial Protocol

**DOI:** 10.1155/2016/2913248

**Published:** 2016-01-28

**Authors:** Kiok Kim, Kyung-Min Shin, Jun-Hwan Lee, Bok-Nam Seo, So-Young Jung, Yousuk Youn, Sang Ho Lee, Jaehong Kim, Wenchun Qu, Tae-Hun Kim

**Affiliations:** ^1^Department of Spine Center, Mokhuri Neck & Back Hospital, Seoul 06272, Republic of Korea; ^2^Clinical Research Division, Korea Institute of Oriental Medicine, Daejeon 34054, Republic of Korea; ^3^Korean Medicine Life Science, University of Science & Technology (UST), Campus of Korea Institute of Oriental Medicine, Daejeon 34113, Republic of Korea; ^4^Mibyeong Research Center, Korea Institute of Oriental Medicine, Daejeon 34054, Republic of Korea; ^5^Department of Physical Medicine and Rehabilitation, Mayo Clinic, Rochester, MN 55905, USA; ^6^Department of Anesthesiology, Division of Pain Medicine, Mayo Clinic, Rochester, MN 55905, USA; ^7^Korean Medicine Clinical Trial Center, Korean Medicine Hospital, Kyung Hee University, Seoul 02447, Republic of Korea

## Abstract

This is a study protocol for a pilot three-armed randomized controlled trial on nonsurgical integrative Korean medicinal treatment for symptomatic lumbar spinal stenosis (LSS). Thirty-six participants who have been diagnosed with (LSS) and recommended for spinal surgery by neurosurgeons or orthopedics and have had spinal symptoms such as severe low back pain and neurological claudication regardless of at least three months of conservative treatments will be recruited. Participants will be randomly assigned to be one of the three intervention groups, including the Mokhuri treatment program group 1 or 2 or usual care group. All treatments will be administered in inpatient units over a period of 4 weeks. The primary outcomes are 0 to 100 Visual Analogue Scales for low back pain and leg pain and the secondary outcomes are Oswestry Disability Index; EQ-5D; Roland-Morris Disability Questionnaire; Oxford Claudication Score; physical function test, including treadmill test, walking duration, and distance assessment for free leg pain; radiologic testing; and adverse events which will be assessed during the 4-week treatment period as well as after 3 and 6 months of follow-up. Then, we will assess the feasibility of the clinical trial design as well as a nonsurgical integrative treatment program. This trial is registered with CRIS registration number: KCT0001218.

## 1. Backgrounds

Lumbar spinal stenosis (LSS) is defined as narrowing of the central canal or neuroforamina, which is identified by radiologic imaging or intraoperational observation [1]. Among several different pathologic causes of LSS, degenerative changes in various structures around the spinal canal or neural foramina including the vertebral disks, facet joints, ligamentum flavum, posterior longitudinal ligament, and lamina are the leading cause of LSS [1]. Based on the Wakayama spine study, the prevalence of LSS is estimated as 10% in all age groups, with a prominent increase in the prevalence reported in those who are over 60 years old, which reflects that LSS is closely related to the degenerative changes [2].

In Korea, no precise information is available on the prevalence of LSS. However, according to the annual report of Health Insurance Review & Assessment Service, approximately one million patients who are diagnosed with LSS visit the hospital; as a result, LSS was ranked as the 56th most frequent cause for all hospital visits in Korea during 2011 [3]. In addition, the total number of hospital admissions for LSS has increased from 11,471 cases in 2000 to 78,372 in 2011 [3].

Spinal fusion surgery and decompression surgery are accepted as the standard treatments for spinal stenosis, and complex fusion surgery involving more than two levels of vertebral disc is more commonly practiced than before [4]. Spinal fusion surgery and decompression surgery were reported as effective during one to two years after surgery, but there is no concrete evidence on the increased effectiveness of these surgeries compared with nonsurgical conservative therapies in the long term [5]. The cost of surgery is considerably higher and there are frequent, severe adverse events related to surgery [4, 6]. In addition, nonsurgical conservative treatments of conventional medicine including drug therapy (acetaminophen, analgesics, and gabapentin), epidural steroid injection, and physiotherapy are used for this condition but high quality evidence on the effectiveness and safety of these nonsurgical treatments is absent either [7].

Currently, the population of seniors over 65 years of age accounts for 11.8% of the entire population, and the medical expenditures of these age groups account for 32.2% of the total expenditure in Korea. The rate of aging in the population is continuously increasing, and the incidence and prevalence of LSS are expected to increase in the future along with the general aging trend. As a result, a considerable increase in the social and economic costs is predicted. Considering this situation, effective and safe interventions are necessary for patients with LSS in Korea [8].

Nonsurgical Korean integrative interventions, including herbal medication, acupuncture, and manual therapy (Chuna), have been used for various spinal conditions, including nonspecific low back pain [9, 10] and lumbar radiculopathy [11]. Currently, however, there is no concrete evidence on the benefits and harms of these nonsurgical, conservative interventions. For spinal stenosis, only observational studies are available [12]; as a result, well-designed clinical trials will be necessary to evaluate the clinical evidence for these interventions.

This study is a pilot clinical trial on nonsurgical Korean integrative treatments for treating LSS. In this study, we will assess the feasibility of the integrated treatment protocol and study design for a randomized, conventional standard treatment, controlled trial for use in a future full-scaled clinical trial.

## 2. Methods

### 2.1. Study Objectives

The purpose of this study is to assess the feasibility of RCTs on clinical effectiveness and safety of the four-week Korean integrative treatment program for severe spinal stenosis, comparing different types of Mokhuri treatment programs and the usual care in conventional medicine in inpatient units. Information related to the compliance of the study design and interventions, the implementation of outcome assessments, and the sample size calculation for the full sized RCTs will be analyzed in this study. The null hypothesis of the primary outcome is that the change of 0-to-100 visual analogue scale (VAS) for low back pain and leg pain before and after treatment will be the same in the following three patient groups: Mokhuri treatment program group 1 (MT1 group) that will be treated with herbal medication, Chuna, acupuncture, and patient education; Mokhuri treatment program group 2 (MT2 group) that will be treated with Chuna, acupuncture, and patient education; or the usual care group that will be treated with analgesics, epidural steroid injection, and physiotherapy.

### 2.2. Methods

This study is a corporative clinical study with Spine Center of Mokhuri Oriental Medical Hospital, Korea Institute of Oriental Medicine, and Complementary and Integrative Medicine Program of Mayo Clinic. The study design was developed through discussion among the three organizations, and the clinical study will be conducted in Mokhuri Hospital. The study protocol was approved by the Institutional Review Board of Mokhuri Hospital (MHNBH-14001). A total of 36 LSS patients with intractable symptoms that did not subside by at least three months of conventional treatment will be recruited through advertisements in newspapers and websites for these patient communities. This is a pilot study for future, full-scaled clinical trials. As a result, 12 patients will be recruited for each group without a sample size calculation for the minimum participant number according to statistical analysis of their basic information, considering possible dropouts. Because patients with both spinal stenosis and spondylolisthesis and therapeutic responses of spinal stenosis with and without spondylolisthesis are expected to differ, we will recruit 2 patients with spondylolisthesis into the 12 participants of each group to assess the difference in the clinical outcomes. The study will be conducted at the Spine Center of Mokhuri Hospital, Seoul, South Korea. In the clinical research information service (CRIS, a representative clinical trials registry platform in Korea), the protocol was registered before the first participant is included (registration number: KCT0001218). Any patients who meet the inclusion criteria and provide written informed consent for participation in the study will be randomly allocated into one of three groups, the MT 1, MT 2, or usual care groups. For sequence generation, stratified random numbers for whether the patients do or do not have spondylolisthesis will be generated using the SAS 9.2 package software. Opaque sealed envelopes will be used to conceal which group patients have been allocated to. Over a period of four weeks, each treatment will be offered in the inpatients unit. Patients will be urged to visit the research center for follow-up evaluation at three and six months after the four weeks of treatments.

### 2.3. Inclusion Criteria

Patients who are aged 19 to 77 years with clinical symptoms and signs consistent with LSS, including low back pain, radicular leg pain, or leg discomfort when standing or walking, will be included in the study. The patients should have experienced claudication or radicular leg symptoms for at least one year and lack symptomatic relief from pain; also, function related to LSS should be experienced after at least three months of sufficient conservative nonsurgical treatments, including epidural injection therapy, administration of analgesics, and (or) physiotherapies. He or she needs to be diagnosed with LSS through CT or MRI and have been recommended for decompression and (or, additionally) fusion surgery by neurosurgeons or orthopedic doctors. Neurogenic claudication should appear within five minutes of walking on a treadmill with a speed of at least 1.5 miles/hour. Finally, the patient should not have been treated with an epidural injection during the last 1 month.

### 2.4. Exclusion Criteria

1. Patients with at least one of the following conditions will be excluded from this study:
  - past or present existence of a movement disorder and orthopedic problems that might affect the ability to ambulate,
  - moderate to severe arthritis of the knee or hip that might severely compromise ambulation,
  - past or present lower extremity peripheral vascular disease or vascular claudication,
  - previous LSS surgery,
  - serious concomitant medical illness (i.e., heart disease, renal failure, and active hepatitis),
  - another specific spinal disorder (e.g., ankylosing spondylitis, infection, metabolic diseases, or severe osteoporosis),
  - presence of major or progressive neurological deficit, including dementia,
  - severe systemic diseases, including coronary artery disease or malignancy,
  - any past or present psychiatric conditions, including depression and others,
  - current usage of narcotic analgesics, including a transdermal patch,
  - present digestive disorders including gastritis, gastric ulcer, and irritable bowel syndrome.
2. Anyone who is currently pregnant or plans to be pregnant will be excluded.
3. Anyone who appears likely to encounter difficulties in adhering to this protocol as a result of noncompliance with treatments (less than 80% of treatment participation), visits, and responding to questionnaires will be excluded.

### 2.5. Intervention

Patients will be allocated into one of three groups, the MT 1, MT 2, or usual care groups, during four weeks in the inpatient unit. Patients in the MT 1 group will receive integrative treatments, including herbal medications, Chuna, acupuncture, and patient education. Those in the MT 2 group will receive Chuna, acupuncture, and patient education without herbal medication. Those in the usual care group will be offered conventional nonsurgical treatments with analgesics, epidural steroid injection, and physiotherapy. Any concomitant therapies for lumbar stenosis will be prohibited during the four-week treatment period in all patients.

In the MT 1 group, herbal medication (Gang-Chuk Tang) will be offered during the participation period. Gang-Chuk Tang (110 g), which is an herbal decoction agent consisting of* Eucommiae Cortex, Achyranthis Radix, Cibotii Rhizoma, Sorbus commixta, Geranium thunbergii, Saposhnikovia Radix*, and* Acanthopanacis Cortex*, will be administered three times a day for four weeks. Chuna is a specific manipulation technique in Korean medicine, which consists of mobilization within the limits of passive range of joint motion and muscle relaxation according to the patient's respiration [13]. In this study, we will perform relaxative Chuna using Ergo style TM FX.-5820 Table (Chattanooga Group, USA); the detailed methods are presented in the following list. 

*Steps for the Chuna Technique*

1. Patients are laid on the table in the prone position. Patients need to relax and breathe slowly.
2. Ergo style COX table is set to work for automatic flexion and distraction of the lumbar vertebra. The flexion angle is adjusted to the patient's condition (within 5 to 15 degrees), and it should not surpass the pain threshold of the patients. The autoflexion speed needs to be adjusted according to the breathing speed.
3. The physician gently pulls and pushes rigid back muscles, including the latissimus dorsi, rhomboid, quadratus lumborum, gluteus medius and minimus, and paraspinal muscles, during autoflexion of the COX table.
4. When the lumbar vertebrae are in flexion, the gaps in the intervertebral spaces can be manually broadened through fixing both hands of the physician at the lamina of the upper vertebra and spinous process of the lower vertebra. The patients should breathe out slowly.
5. Steps (3) and (4) continue from the lumbar vertebra to thoracic vertebra.
6. General lumbar roll manipulation is applied in both sides of the lumbar vertebra. Patients need to be laid on the table in the lateral recumbent position.

Patients will attend approximately ten minutes of Chuna treatment. For acupuncture therapy, 0.25 × 40 mm disposable stainless steel needles will be used. LI4, ST36, LV3, BL22, BL23, BL24, BL25, and Ashi points will be selected for acupuncture. The depth of insertion will be 1.5 to 30 mm for each point applied to induce “deqi sensation.” Acupuncture needles will be retained for approximately 15 minutes. A certified Korean medicinal doctor who has over 10 years of clinical experience will offer Chuna and acupuncture treatment five times per week over a period of four weeks. Patient education, which consists of teaching precautions related to daily activity and walking training methods, will be conducted through face-to-face counseling with the physician five times a week over a period of four weeks. The patients will be offered standard education information (see the following list).

*Patient Education Information*

*General Notes*

1. The patients are to relax in bed, because of the possibility that they will experience symptoms in the incubation period that they might not feel prior to the treatment during the first week of treatment.
2. From the second week of the treatment, the patients must practice simple movements such as moving the lower limbs, head, or shoulders slightly to the left and to the right according to the instructions of the medical team. In particular, it will be necessary to compare the symptoms patients experience for a week of treatment on a weekly basis. In addition, the patients should be able to accurately compare and be aware of their neurologic claudication and pain as well as that experienced during the early morning period of relieving tension.
3. In cases of maladaptive treatment-related symptoms (e.g., extreme pain continuing for more than a month that does not respond to appropriate medications, disorders of urinary and fecal continence, and sudden loss of function or sensation in the lower limb), it will be necessary to proceed to surgical intervention to prevent secondary neurological damage.

*Cautionary Notes regarding Walking Training or Routine Walking *

1. Walking time is to be adjusted according to the conditions of the patient. However, adjustments will be required in the walking time from 10 to 20 minutes and thus patients may need to rest by suspending the exercise in cases of pain.
2. During the first week of treatment, patients may experience symptoms in the incubation period and hence may need to rest by limiting their exercise level to the minimum.
3. During the second week of treatment, the patients shall increase their walking distance for 20 m after two to three days depending on each patient's individual situation.
4. After the end of treatment sessions, the patients must increase their walking time by one minute every three days for two to three months and must aim to walk routinely for approximately one half hour without pain.

*Cautionary Notes for Exercise*

1. Intensive exercise must be avoided. Excessive rotational movement of the lumbar or cervical vertebrae increases the load onto slipped disks, leading to further pain exacerbation. Therefore, the exercise regimen must be properly adjusted according to the orders of the medical team.
2. The lumbar and cervical vertebral exercises conducted by our hospital shall be performed via a predetermined frequency (moving the head to the left and the right, shrugging the shoulders, and exercising the lower limb, 20 repetitions for 5 sets). Care must be taken not to excessively increase the repetitions.

*Notes regarding Movements in Activities of Daily Living*

1. When lifting objects, use the knees as much as possible. Patients should avoid lifting excessively heavy objects and should also avoid performing work in a crouched position.
2. During the treatment period and for two to three months after the treatment sessions, patients shall limit their sitting time (not more than 10 minutes at a time). Excessive strenuous exercise shall be avoided.
3. In circumstances wherein patients must maintain a seated position, such as when eating a meal, patients must avoid an Indian-style seated position and must relieve the burden on the lumbar region by having the legs out of bed.

*Notes regarding Work*

1. The work load must be reduced as much as possible during the treatment period. In cases requiring the unavoidable processing of work duties, the time devoted to computer use must be reduced.
2. The use of electronic devices such as computers or smart phones for long periods may cause pain in the cervical vertebral and lumbar areas. Therefore, the use of these devices must be avoided as much as possible.

Patients in the MT 1 group will be offered an herbal decoction, Chuna, acupuncture therapy, and patient education, while those in the MT 2 group will be offered Chuna, acupuncture therapy, and patient education during the treatment period.

In the usual care groups, conventional conservative treatments for LSS will be offered [14]. Drugs, including muscle relaxants, NSAIDs, gabapentin, and limaprost, will be prescribed by the neurosurgeon and will be continued during the four weeks. Epidural steroid injections will be offered three to four times in the inpatient unit. Physiotherapy, including 10 to 20 minutes of treatment with a heating pad (STEMKOREA Co., Korea), transcutaneous electrical nerve stimulator (TENS, Homer Ion Laboratory Co. Ltd., Japan), and deep tissue heating therapy (Radio Derm, RoboMax Co., Korea), will be applied at the affected region once a day for five days a week over a period of four weeks. A neurosurgeon will prescribe medications and inject patients with an epidural steroid injection for symptom relief. A physiotherapist will conduct the physiotherapy.

### 2.6. Outcomes

The primary outcomes are 0-to-100 VAS for low back and leg pain. The secondary outcomes are Oswestry Disability Index (ODI); EQ-5D; Roland-Morris Disability Questionnaire (RMDQ); Oxford Claudication Score (OCS); physical function tests, including treadmill tests, assessment of the walking duration, and distance without leg pain; radiologic testing; and adverse events. Every outcome variable will be assessed before and after treatment, and follow-up will continue for up to 6 months in all groups (Table 1).

Zero-to-100 VAS for average low back and leg pain during last week will be assessed once a week during the participating treatments. Arithmetic mean of the peak pain and lowest pain, which the patient experienced during last week regardless of position and activity (walking/standing), will be used as the average VAS score for low back and leg pain. If the patient has pain in both legs, the scores from the more severe side will be recorded. ODI is a widely used tool for assessing the disability related to low back pain. It consists of ten dimensions, such as the pain severity, ability for self-care, lifting, walking, standing, sitting, traveling, sexual function, and sleep quality. Each dimension can have 1 to 6 scores and the ODI can be calculated through the total sum of scores multiplied by 100 and divided by the numbers of answered dimensions. In addition, individual scores of 10 items will be assessed as well. The validated Korean version of ODI will be used in this study [15]. The EQ-5D will be evaluated to assess the quality of life; it has five questions that assess the respondent's mobility, self-care, daily activities, pain, and anxiety/depression, which can have scores of 1 to 3. Patients will have a five-digit number combination, which represents the quality of life. A validated Korean EQ-5D will be used for this study [16]. RMDQ is a tool for assessing the functional status of patients with low back pain. It has 24 questions, and each has yes/no options. The RMDQ score is calculated through adding all questions that are checked as yes so that it ranges from 0 (no functional limitation) to 24 (worst condition). The validated Korean version will be used for this study [17]. The OCS is a tool for evaluating the claudication severity and leg symptoms, which are the most bothersome problems for the patients with LSS. Ten questions related to three dimensions, including pain, ischemic symptoms, and physical symptoms, can have a score of zero (no symptoms) to 5 (worst symptoms), and they can be presented with the percentage of the possible maximum score. Because the RMDQ does not have validated Korean version, we will use a tentatively translated version [18]. To assess the physical function of the patients, the treadmill and walking tests will be evaluated. In this treadmill test, patients walk on a flat treadmill at a speed of 1.5 miles/sec until they complain of leg pain. For the walking test, patients will be ordered to walk on a 50 m flat track at a comfortable pace, and the time until leg pain starts will be recorded. Radiologic testing, including L-spine MRI and X-ray flexion and extension views, will be conducted to evaluate the condition of the spinal canal with stenosis and lumbar spine instability. For patients with spondylolisthesis, changes in the spinal instability will be evaluated. In addition, the AP diameters between pre- and posttreatment will be evaluated to check on the structural changes. The types and frequency of additional treatments usage during follow-up period will be assessed and analyzed after the end of the study. Adverse events (AEs) related to treatments will be monitored by the physicians, and patients should report any unexpected signs or symptoms during the treatment. AEs will be appraised using Spilker's classification, and the types and frequency will be analyzed. Outcome assessors will be open to the allocation results of the participants.

### 2.7. Feasibility Assessment

Recruitment rate and dropout rate during the study period will be assessed for evaluating the feasibility of the study design. Reasons for the dropouts will be analyzed. Compliance to the offered treatment interventions and outcome assessment will be evaluated based on the compliance rate of participants as well as qualitative analysis on the outcome assessors' experiences. For future full-scaled RCT, we will calculate sample size based on the study result.

### 2.8. Statistical Analysis

Statistical analysis will be conducted on the basis of intention-to-treat analysis (ITT). Missing values will be imputed by the last observation carried forward (LOCF) method. Baseline characteristics will be presented as the mean and standard deviation (SD) for continuous outcomes and as the frequency for categorical outcomes. Statistical analysis between groups will be conducted with a *t*-test (Wilcoxon rank sum test in case of nonnormal distribution data) or a chi-square test, respectively. Primary and secondary outcomes will be analyzed with analysis of covariance (ANCOVA), with the change in the outcome scores between the groups as the dependent variable, baseline scores as a covariate, and study groups as a fixed factor. Pre- and posttreatment differences in each group will be analyzed with a *t*-test (Wilcoxon rank sum test) or a chi-square test. Repeated measures analysis of variance will be conducted to identify the trend in data. The frequency of AEs in each group will be compared using Fisher's exact test. The types of AEs will be assessed through qualitative analysis.

### 2.9. Data Monitoring and Dropout Criteria

Data monitoring will be conducted regularly by researchers from the Korea Institute of Oriental Medicine for fair clinical trial. Source data from the study will be handled and analyzed by an independent statistician. Patients who want to stop participating in this study, do not complete the treatment over four visits, violate the study protocol, withdraw consent for participation, and use prohibited treatments for lumbar stenosis will be dropped out from the study.

## 3. Discussion

This is a protocol for a pilot clinical trial on integrative Korean medicinal treatment for symptomatic spinal stenosis. Considering that the aging population is expected to increase, more people will suffer from LSS in the future. Spinal fusion and decompression surgeries, which are the standard treatment for symptomatic LSS, cannot ensure a long-term effect, and adverse events related to the surgery cannot be ignored. In this sense, the currently used nonsurgical treatments need to be evaluated. The findings of this pilot study will be used for the assessment of the feasibility about the study design and compliance of the interventions and outcome assessments, which can be basic data for subsequent full-scale clinical trial on the benefits and harm of this integrative Korean medicinal treatment program for symptomatic spinal stenosis.

## Figures and Tables

**Table 1:** Schedule for outcome assessment.

Stage	Screening	Treatment period in the inpatient unit				Follow-up		
Weeks	0	2	3	4	5	6	18	30
0-to-100 visual analogue scales for low back and leg pain		◯	◯	◯	◯	◯	◯	◯
EQ-5D		◯	◯	◯	◯	◯	◯	◯
Oswestry Disability Index		◯	◯	◯	◯	◯	◯	◯
Roland-Morris Disability Questionnaire		◯				◯	◯	◯
Oxford Claudication Score		◯				◯	◯	◯
Walking duration and distance without pain	◯	◯	◯	◯	◯	◯	◯	◯
Radiologic test (L-spine X-ray of flexion and extension views and L-spine MRI)	◯					*⨀*		*⨀*
Adverse events		◯	◯	◯	◯	◯	◯	◯

◯: outcome variables that will be assessed in all participants; *⨀*: L-spine X-ray of flexion and extension views will only be assessed in the patients with spondylolisthesis.
